# Performance of 4Kscore as a Reflex Test to Prostate-specific Antigen in the GÖTEBORG-2 Prostate Cancer Screening Trial

**DOI:** 10.1016/j.eururo.2024.04.037

**Published:** 2024-05-20

**Authors:** Andreas Josefsson, Marianne Månsson, Kimia Kohestani, Vasiliki Spyratou, Jonas Wallström, Mikael Hellström, Hans Lilja, Andrew Vickers, Sigrid V. Carlsson, Rebecka Godtman, Jonas Hugosson

**Affiliations:** a Department of Urology, Institute of Clinical Sciences, Sahlgrenska Academy, University of Gothenburg, Gothenburg, Sweden; b Wallenberg Center for Molecular Medicine, Umeå University, Umeå, Sweden; c Department of Urology and Andrology, Institute of Surgery and Perioperative Sciences, Umeå University, Umeå, Sweden; d Department of Radiology, Institute of Clinical Sciences, Sahlgrenska Academy, University of Gothenburg, Gothenburg, Sweden; e Department of Radiology, Sahlgrenska University Hospital, Gothenburg, Sweden; f Department of Pathology and Laboratory Medicine, Memorial Sloan Kettering Cancer Center, New York, NY, USA; g Department of Surgery, Memorial Sloan Kettering Cancer Center, New York, NY, USA; h Department of Medicine, Memorial Sloan Kettering Cancer Center, New York, NY, USA; i Department of Translational Medicine, Lund University, Malmö, Sweden; j Department of Epidemiology and Biostatistics, Memorial Sloan Kettering Cancer Center, New York, NY, USA; k Department of Translational Medicine, Division of Urological Cancers, Medical Faculty, Lund University, Skåne University Hospital, Malmö, Sweden

**Keywords:** Prostate cancer, Screening, 4Kscore, Prostate-specific antigen, Magnetic resonance imaging, GOTEBORG-2 study

## Abstract

**Background and objective:**

We investigated whether adding 4Kscore as a reflex test to prostate-specific antigen (PSA) could improve the screening algorithm for prostate cancer (PC).

**Methods:**

In the GÖTEBORG-2 PC screening trial, 38 000men (50–60 yr) were invited to PSA testing and, if elevated, followed by magnetic resonance imaging (MRI). For 571 men with PSA ≥3.0 ng/ml and evaluable outcomes, 4Kscore was calculated. The performance using a prespecified 4Kscore cutoff of 7.5% was evaluated.

**Key findings and limitations:**

The area under the curve for 4Kscore to identify intermediate- and high-risk PC was 0.84 (95% confidence interval 0.79–0.89), and the positive predictive value, and negative predictive value were 15% (0.12–0.20) and 99% (97–100%), respectively. Of the 54 men diagnosed with intermediate- or high-grade PC, two had a 4Kscore cutoff below 7.5%, both with organ-confined intermediate-risk PC. Per 1000 men with elevated PSA, adding 4Kscore would have resulted in avoidance of MRI for 408 (41%) men, biopsies for 95 (28% reduction) men, and diagnosis of 23 low-grade cancers (23% reduction) while delaying the diagnosis of four men with intermediate-grade cancers (4%).

**Conclusions and clinical implications:**

Including 4Kscore as a reflex test for men with elevated PSA reduces the need for MRI and biopsy markedly, and results in less overdiagnosis of low-grade PC at the cost of delaying the diagnosis of intermediate-grade PC in a few men. These results add further evidence for including new blood-based biomarkers in addition to PSA to improve the harm and benefit ratio of PC screening and reduce the need for resource-demanding MRI and biopsies.

## 1. Introduction

Screening with prostate-specific antigen (PSA) followed by systematic biopsies has been shown to decrease prostate cancer mortality but is also associated with several harms [1–3]. First, PSA has low specificity, resulting in many men undergoing biopsies; three out of four men with elevated PSA have no cancer. Second, many cancers detected by systematic biopsies are small, low-grade, International Society of Urological Pathology grade 1 (ISUP 1), tumors with a very low risk of progression [4]. This overdiagnosis of clinically “insignificant” cancers has been estimated to constitute 40–60% of all cancers detected by PSA followed by systematic biopsies [2,3]. This high rate of overdiagnosis has been the main argument against introducing population-based screening with PSA. During recent years, utilization of magnetic resonance imaging (MRI) in men with elevated PSA has been shown to identify suspected tumor lesions in the prostate, and by targeting biopsies toward suspected tumor areas instead of systematic biopsies, there is a decreased risk of overdiagnosis of low-grade, ISUP 1 prostate cancers [5,6]. These findings led the European Union to change its position in favor of prostate cancer screening last year [7]. The Union now recommends member states to evaluate the feasibility and effectiveness of organized prostate cancer screening.

MRI is a resource-dependent service, and access to MRI equipment and experienced radiologists are bottlenecks in a health care system for organized prostate cancer screening. A biomarker that could replace or complement PSA with improved positive (PPV) and negative (NPV) predictive values to rule in intermediate- to high-grade cancers (ISUP 2–5) [4] but rule out benign lesions as well as low-grade cancers is warranted and would consequently decrease the number of men needing MRI. Several such biomarkers are available, but few studies have investigated their utility in a screening setting to triage men before MRI and biopsy [8–11].

In this study, designed as a blinded biomarker investigation based on a predefined statistical analysis plan, we prospectively invited men from the GÖTEBORG-2 (G2) [6] trial to also participate in correlative biomarker studies based on the cryopreservation of whole blood, plasma, and serum. Our study question is whether the use of 4Kscore as a reflex text after an elevated PSA level, but before referral to MRI, would reduce the burden of further diagnostic workup and overdiagnosis of low-grade prostate cancer without delaying the diagnosis of an undue number of intermediate- or high-grade cancers.

## 2. Patients and methods

### 2.1. Trial design and participants

The GÖTEBORG-2 biomarker (G2B) study is a biomarker study embedded in the G2 trial, which has been described in detail previously [6,12]. In brief, approximately 38 000 men (50–60 yr) were randomized to a screening group identified from the Swedish Population Register and invited to PSA screening. Men who agreed to participate by having a PSA test were allocated to one of three trial arms. In all three arms, four targeted biopsies against that region with the cognitive fusion technique were obtained from each MRI-positive lesion defined as any Prostate Imaging Reporting and Data System (PI-RADS) ≥3 lesion using cognitive MRI–transrectal-ultrasound fusion technique (see also the Supplementary material).

The G2B study was approved by the regional ethical board (988-15 and T294-18) as an auxiliary study to G2. Participants in the G2 trial with elevated PSA received, together with the MRI invitation, a letter with detailed written information about the G2B study. Men who accepted to participate in the trial signed the informed consent form prior to blood draw for biobanking. Owing to logistical challenges with biobanking and the limited availability of a study nurse, especially during the height of the COVID-19 pandemic in 2020–2022, inclusion was limited; hence, not all eligible men were offered inclusion.

Men eligible for this study were all men enrolled in the G2 study until February 15, 2020, with a PSA level of ≥3.0 ng/ml, who had evaluable MRI and who accepted inclusion after providing signed written informed consent in the G2B study. Men who had positive MRI (PI-RADS ≥3) but declined to undergo a biopsy were excluded from the analysis. Only men with positive MRI underwent targeted biopsy. For laboratory measurements and pathological assessment, see the Supplementary material.

### 2.2. Statistical analysis

The primary objective was to assess the performance of 4Kscore as a reflex test among men with elevated PSA to select men who would need further evaluation with MRI and potential biopsies. The 4Kscore calculation was performed according to a prespecified algorithm [13] and prespecified cutoff [14].

For the prespecified cutoff of 4Kscore of 7.5%, NPV and PPV for the detection of intermediate- to high-grade cancer were estimated with 95% confidence intervals (CIs). Only MRI-targeted biopsies were taken into account in this study. Hence, these estimates are relative to cancers found in MRIpositive men, and not in all men. To illustrate the clinical consequences of adding 4Kscore as a reflex test before MRI in the G2 study algorithm, the numbers of spared MRI scans, biopsies, and prostate cancers caught and missed per 1000 men were estimated for the prespecified cutoff of 7.5%. As a post hoc analysis, we have also analyzed the 4Kscore cutoff of 5.0% (Supplementary material).

The predictive accuracy of 4Kscore as a reflex test was evaluated by means of the area under the curve (AUC) and a decision curve analysis. The statistical analyses were performed in R Statistical Software, version 4.2.3. The statistical analysis plan was set before any analysis of the data (Supplementary material).

## 3. Results

**Fig. 1 –:**
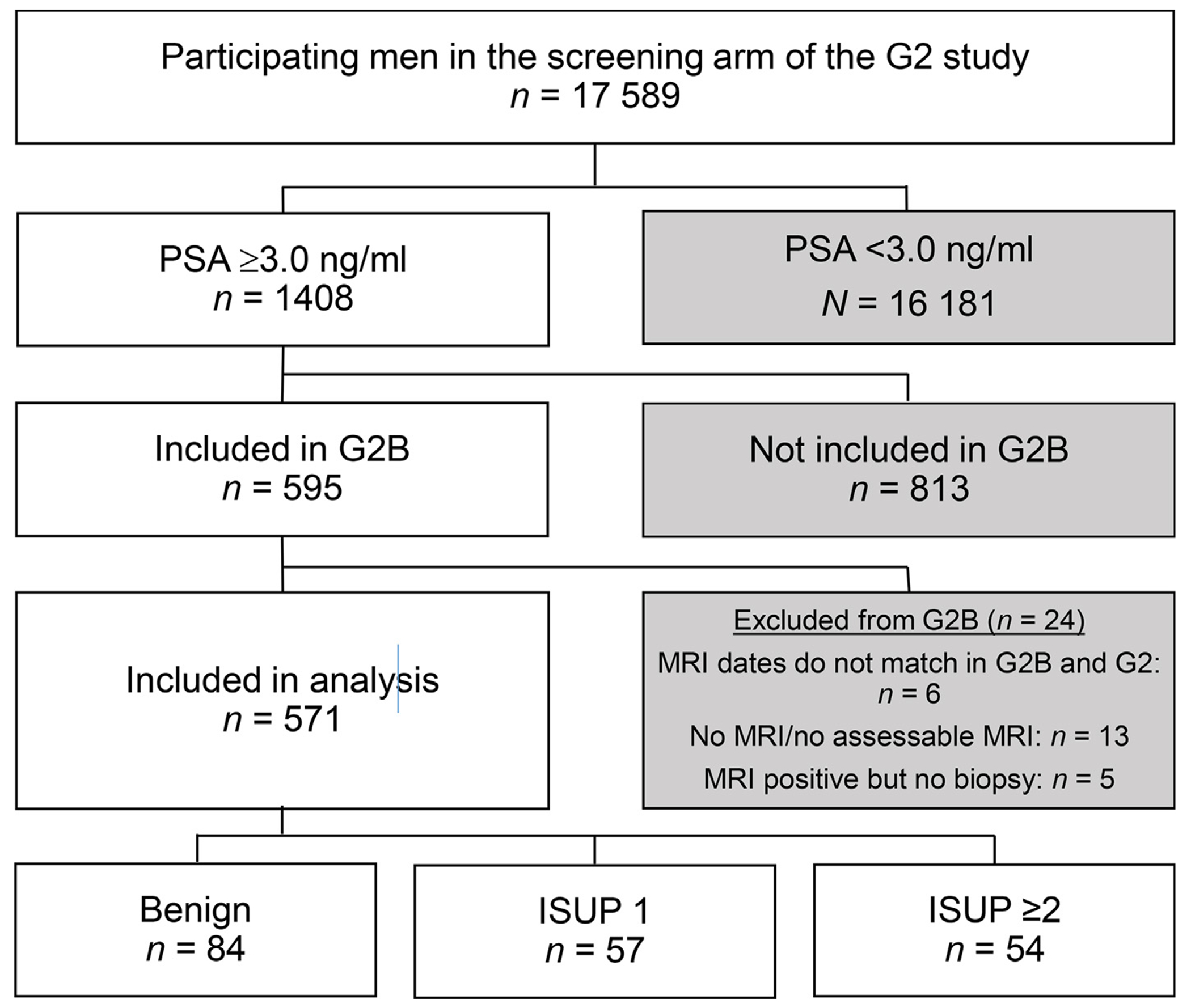
Study flow. G2 study = GÖTEBORG-2 randomized screening study; G2B = biomarker study within G2 study; ISUP = International Society of Urological Pathology; MRI = magnetic resonance imaging; PSA = prostate-specific antigen.

By February 2020, 17 589 men had participated in G2 by having a PSA test, and of them, 2116 were invited to undergo an MRI examination. In total, 932 men were enrolled in the G2B study, in which 595 had a PSA level of ≥3.0 ng/ml. Of these individuals, 24 were excluded (Fig. 1). Thus, 571 men remained for analyses. Of these men, 42 had participated in the G2 screening study at least once before inclusion in G2B; of them, 32 had undergone MRI and 15 undergone biopsies with benign outcomes (Fig. 1).

There were no apparent differences in baseline or outcome characteristics between men included in the G2B study and those who were not included (Table 1). The median age of the G2B participants was 59 yr, and 49% had PSA levels between 3.0 and 3.99 ng/ml, 46% had PSA levels between 4.0 and 9.99 ng/ml, and 6% had PSA levels of ≥10.0 ng/ml (Table 1).

Approximately one-third of the men (34%, 195/571) had positive MRI (PI-RADS 3–5), and the pathology results from the subsequent targeted biopsies were as follows: benign 43%, low-grade cancer 29%, and intermediate- to high-grade cancer 28% (Table 1).

**Fig. 2 –:**
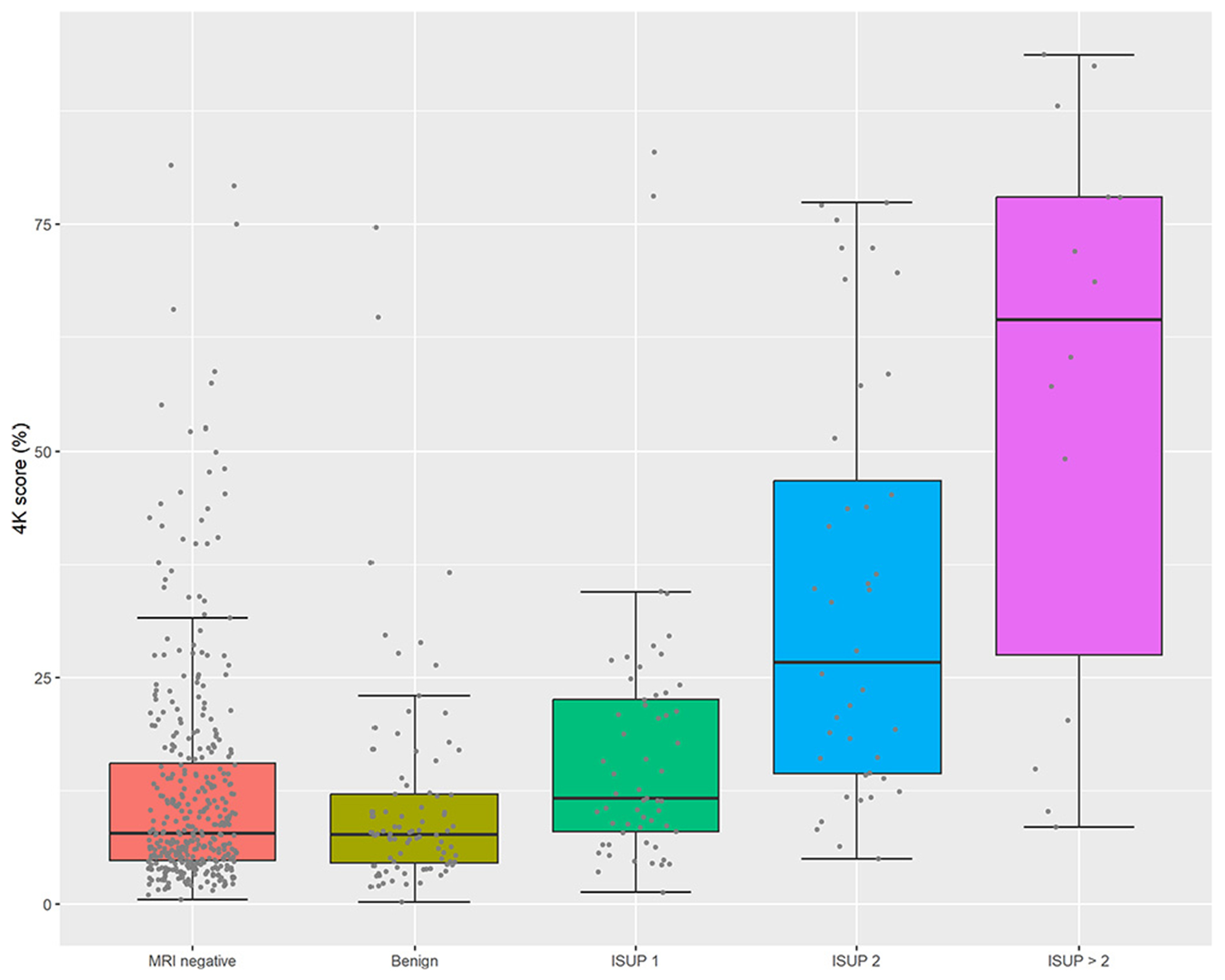
Box-dot plots of 4Kscore distribution by MRI and biopsy outcome. MRI = magnetic resonance imaging; ISUP = International Society of Urological Pathology; MRI = magnetic resonance imaging.

4Kscore cutoffs are shown in Figure 2. Of the men, 23% (130/571) had a score of <5%, 18% (103/571) had a score between 5% and 7.5%, 12% (69/571) had a score between 7.5% and 10%, and 47% (269/571) had a score of >10%.

**Fig. 3 –:**
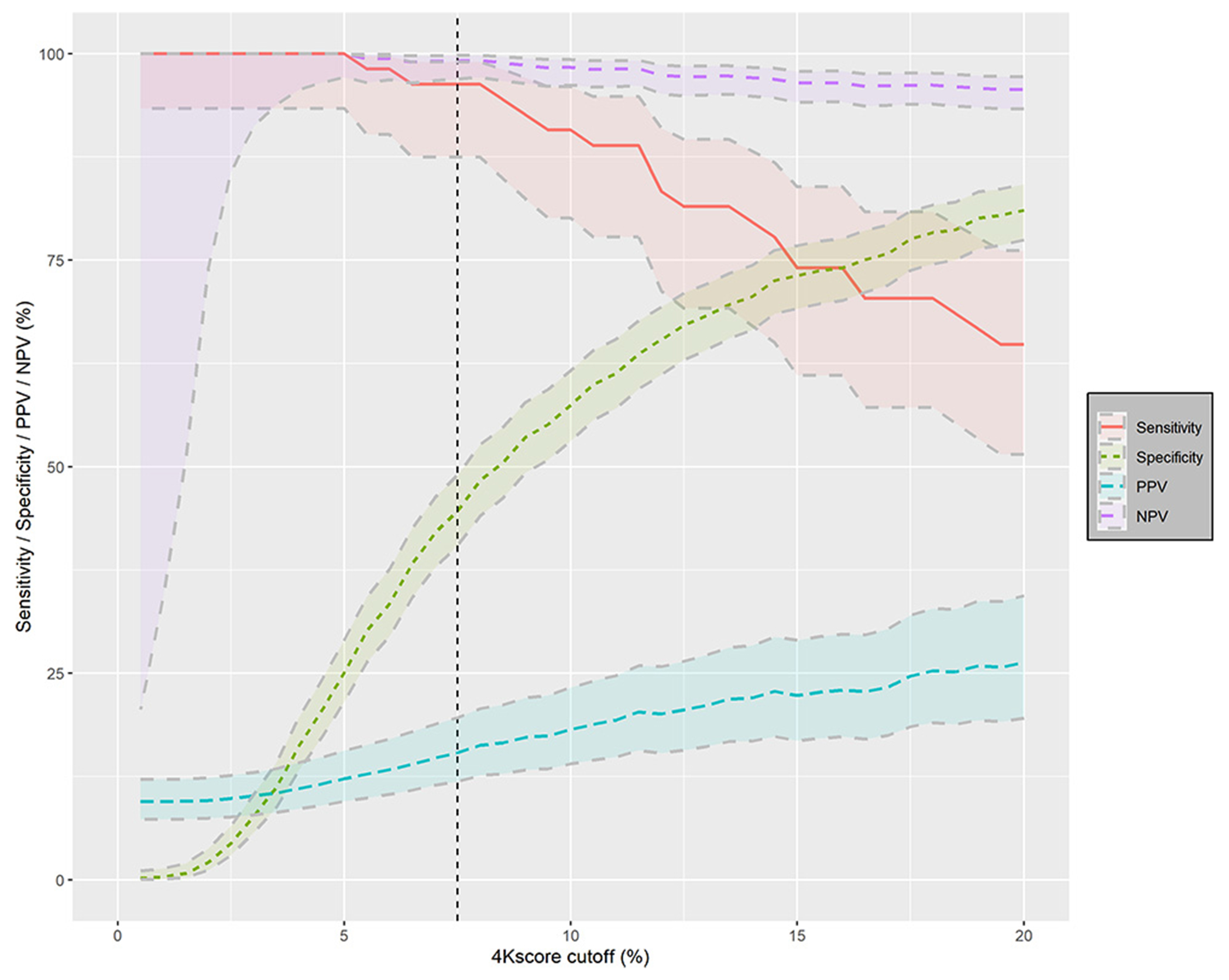
Sensitivity, specificity, PPV, and NPV by different cutoffs of 4Kscore for the diagnosis of intermediate- to high-grade prostate cancer (ISUP ≥2). ISUP = International Society of Urological Pathology; NPV = negative predictive value; PPV = positive predictive value.

The PPVs of intermediate- to high-grade prostate cancer (ISUP ≥2) using a 4Kscore cutoff of ≥7.5%, along with their corresponding 95% CIs, were 15% (12–20%), and the NPVs were 99% (97–100%). For the purposes of comparison with other markers in the literature, sensitivity was 96% (95% CI 87–99%) and specificity 45% (95% CI 40–49%). Figure 3 shows the test performance of 4Kscore cutoffs of up to 20% with PPV, NPV, sensitivity, and specificity for the detection of ISUP ≥2 PC (Fig. 3; forany-grade cancer, see Supplementary Fig. 1). The performance of 4Kscore was better for intermediate- to high-grade cancer than for any-grade cancer.

Of the 54 men diagnosed with intermediate- or high-grade prostate cancer, only two men had a 4Kscore cutoff of ≥7.5%, and both men had PSA levels in the range between 3 and 4 ng/ml. Both men had 4Kscore cutoffs between 5.0% and 7.5%, and targeted biopsies demonstrated organ-confined ISUP 2 cancers with PSA levels of 3.1 and 3.4 ng/ ml, respectively.

Table 2 shows the clinical implications of using 4Kscore as a reflex test. Using a 4Kscore cutoff of ≥7.5%, the estimated number of avoided MRI scans would be 408 (41%) per 1000 men with elevated PSA, and 95 (28%) biopsies could have been avoided. By implementing this cutoff, diagnosis of low-grade cancers (ISUP 1) would have been reduced by 23 (23%) cases. The analysis results of the 4Kscore cutoff of 5.0% are presented in Supplementary Table 3.

The AUC for the 4Kscore as a reflex test before MRI was calculated to be 0.84 (95% CI 0.79–0.89) for intermediate- to high-grade prostate cancer and 0.75 (95% CI 0.70–0.80) for any-grade cancer. A decision curve analysis showed that including 4Kscore had a higher net benefit than performing MRI on all or none for threshold probabilities above 3% (Supplementary Fig. 2).

Cross-tabulation of 4Kscore intervals versus pathology results for men with positive MRI show that the proportion of benign (unnecessary) biopsies varied from 77% in the 4Kscore interval of 0–5% to 25% if the 4Kscore cutoff was above 10% (Supplementary Table 1). A similar cross-tabulation of 4Kscore and MRI results demonstrated that 23 of the 25 men with a PI-RADS 5 lesion on MRI had a 4Kscore cutoff of above 10%, whereas 58% of all men with a 4Kscore cutoff of above 10% had negative MRI and therefore had no MRI-targeted biopsies (Supplementary Table 2).

## 4. Discussion

We found that the use of the 4Kscore threshold of ≥7.5% as a reflex test in men with PSA ≥3.0 ng/ml would reduce the number of MRI scans needed by 41%, and 28% of men would be spared a biopsy. Detection of low-grade prostate cancer would be reduced by 23% at the cost of delaying the diagnosis of intermediate- to high-grade prostate cancer in very few men.

The introduction of prostate MRI has changed the diagnostic pathway for prostate cancer dramatically. Today, most guidelines recommend prebiopsy MRI in the workup of men with elevated PSA [15–17]. Recently published data from randomized screening trials have demonstrated that the PSA-MRI pathway works well in populations of asymptomatic middle-aged men invited to screening. In the first analysis from the G2 trial, overdiagnosis was reduced by half by performing MRI-directed targeted biopsies instead of systematic biopsies [6].

We tested the value of 4Kscore in an MRI pathway, so the performance characteristics we report here are only valid for that pathway. Performance characteristics of 4Kscore in other pathways, for instance, without MRI or with a prior secondary marker, cannot be derived from our findings here. That is, the sensitivity is based on MRI-positive men with cancer, while the specificity is based on either MRI-negative or MRI-positive men without cancer, and similarly for PPV and NPV.

Only two men had intermediate- to high-grade prostate cancer with a 4Kscore cutoff of <7.5%. As demonstrated in the decision curve analysis, net MRI scans avoided already started at a risk threshold of 3%. It is possible to calculate 4Kscore with or without adding clinical data. Another advantage of 4Kscore is that it can be used as a true reflex test, based on the same blood sample collected for PSA testing, and then analyzed only in the subgroup of men with elevated PSA, not requiring any clinical data such as family history or prostate volume.

The performance of 4Kscore is currently evaluated prospectively in the ProScreen trial in Finland, a population-based randomized screening trial investigating a three-step screening strategy combining PSA, 4Kscore, and MRI [18]. Results from the ProScreen pilot, which invited 400 men aged 64–65 yr, were published recently [19]. Out of 27 men with PSA ≥3.0 ng/ml, MRI could be spared in five (19%) due to a 4Kscore cutoff of <7.5%. In comparison, 28% of biopsies could be spared in the current study. The performance of 4Kscore has previously been evaluated in several different settings and has been shown to improve the detection of high-grade prostate cancer in both European and US cohorts [14,20–22]. The study by de la Calle et al [22] presented data on 4Kscore performance in 783 men, but in contrast to our study, it used the 4Kscore in the decision-making on whether biopsies were performed or not, which might introduce a verification bias. Moreover, 4Kscore can predict short-term outcomes, such as the risk of adverse pathology and PSA recurrence after radical pathology, but also longer-term outcomes, such as prostate cancer metastasis and death [23,24]. Critically, the 7.5% threshold used in this study was derived and validated in studies of men not subject to screening and followed for many years. Men at age 60 yr with a PSA level of ≥3.0 ng/ml and a 4Kscore cutoff of <7.5% had zero and 0.55% risks of prostate cancer death within 10 yr [23], which, given the lead time of prostate cancer screening, means that biopsy can safely be avoided in these men.

Several other potential blood-based biomarkers are available commercially, but few have been investigated in a screening setting [25]. One exception is the Stockholm 3 test, which combines protein markers, genetic markers, and clinical data to assess an individual’s prostate cancer risk. In the STHLM 3 MRI study, the Stockholm 3 test, followed by MRI-targeted biopsies, was compared with traditional approaches for prostate cancer screening in a randomized population-based study. The use of the Stockholm 3 test with a cutoff of 0.15% as a trigger for MRI instead of PSA ≥3.0 ng/ml decreased the number of MRI scans needed by 36% [11]. This figure is similar to the 41% reduction we observed for 4Kscore. The reduction in the number of biopsies and low-grade prostate cancer diagnoses was lower than reported herein, 8% and 11%, respectively, for the Stockholm 3 test, compared with 28% and 23%, respectively, for the 4Kscore test. The performances of individual biomarkers across different study populations are difficult to compare directly. However, the key point is that the STHLM 3 MRI study gives support to the general strategy of using a marker as a reflex test for elevated PSA before MRI. The advantages of this strategy, in comparison with an MRI-first approach, have been discussed previously [26]. In particular, the use of markers as a reflex test has considerable advantages in terms of anxiety—men are not told that they are at risk of cancer and need a scan—and convenience, with the test run from the same blood sample, obviating the need for an additional visit. However, health economic evaluations are still lacking and are needed before any additional biomarker panel can be recommended in clinical guidelines.

The present study’s main strength is that we evaluated the performance of 4Kscore in a population-based cohort of men randomized from the general population. Men were asked to participate based on the availability of a study nurse to also collect blood samples for the G2B study, limiting the inclusion but reasonably maintaining a random selection of participants. Baseline characteristics of the G2B population were similar to those of the men in the G2 study who were not enrolled. Another strength is that MRI scans and biopsies have been performed and evaluated in standardized ways, strictly defined in a study protocol, by experienced specialists.

The main limitation is that we primarily report data from a single screen; only 42 men had previously participated once or twice in the screening study. That said, 4Kscore has been expressly evaluated in men with prior screening [27] and in the US validation study [13], most men had prior PSA tests. A large proportion of men with elevated 4Kscore cutoffs had negative MRI scans and have, therefore, not been biopsied, and repeated screening rounds and longer follow-ups are needed to clarify the clinical significance of these elevated scores. However, the accumulating evidence that MRI-negative men have a very low risk of developing clinically significant prostate cancer supports this approach for our endpoint in the study. The present study design cannot determine the optimal PSA threshold for adding 4Kscore. It is well known that clinically significant cancers are present, as well as PSA levels of <3.0 ng/ml. The 4Kscore measurements were performed blinded to outcome using cryopreserved blood obtained at MRI and separate from the PSA at screening. Theoretically, the results could be different when 4Kscore is measured as a reflex test using the same blood sample as the primary screening test (PSA), although this is not likely.

## 5. Conclusions

Including 4Kscore as a reflex test for men with elevated PSA would importantly reduce the need for MRI and biopsy, leading to decreased overdiagnosis of low-grade cancer at the cost of delaying diagnosis of intermediate-grade prostate cancer in only a few men. Comparative studies of available biomarker panels, including health economics, are needed to evaluate their relative performance and determine their optimal role in future screening approaches.

## Supplementary Material

1

2

## Figures and Tables

**Table 1 –:** Baseline characteristics of men included in the G2B study with PSA >3.0 ng/ml with evaluated outcome of MRI, with targeted biopsies, and participated in the G2B study or not

	G2B participants	G2B nonparticipants
	(*n* = 571)	(*n* = 742)
Age, median (IQR)	59 (56, 61)	59 (55, 61)
PSA interval (ng/ml), *n* (%)		
3.0–<4.0	278 (49)	377 (51)
4.0–<10.0	260 (46)	325 (44)
≥10.0	33 (5.8)	40 (5.4)
PSAD interval, *n* (%)		
<0.1	348 (61)	427 (58)
0.1–<0.15	148 (26)	192 (26)
≥0.15	75 (13)	120 (16)
NA	0 (0.0)	3 (0.4)
PI-RADS, *n* (%)		
1	7 (1.2)	12 (1.6)
2	369 (65)	501 (68)
3	53 (9.3)	51 (6.9)
4	117 (21)	133 (18)
5	25 (4.4)	45 (6.1)
MRI result, *n* (%)		
Negative	376 (66)	513 (69)
Positive	195 (34)	229 (31)
Biopsy results, *n* (%)		
Benign	84 (43)	75 (33)
ISUP 1	57 (29)	81 (35)
ISUP 2	40 (21)	49 (21)
ISUP >2	14 (7.2)	24 (11)

G2B study = biomarker study within GÖTEBORG-2 randomized screening study; IQR = interquartile range; ISUP = International Society of Urological Pathology; MRI = magnetic resonance imaging; NA = not available; PSA = prostate-specific antigen; PSAD = PSA density; PI-RADS = Prostate Imaging Reporting and Data System.

**Table 2 –:** Detection rates of cancers and saved MRI scans and biopsies per 1000 men with elevated PSA, using 4Kscore with a cutoff of 7.5% as a reflex test in the diagnostic workup for men with PSA ≥3.0 ng/ml followed by MRI and targeted biopsies of PI-RADS score ≥3

	PSA ≥3.0 ng/ml as trigger for diagnostic workup Performed	PSA ≥3.0 ng/ml and 4Kscore ≥7.5% for diagnostic workup	
		Performed	Avoided/missed, *n* (%)
MRI	1000	592	408 (41)
Negative MRI	658	345	312 (48)
Positive MRI (PI-RADS ≥3)	342	247	95 (28)
Targeted biopsies	342	247	95 (28)
Benign biopsies	147	79	68 (46)
ISUP 1	100	77	23 (23)
ISUP ≥2	95	91	4 (4)

ISUP = International Society of Urological Pathology; MRI = magnetic resonance imaging; PI-RADS = Prostate Imaging Reporting and Data System; PSA = prostate-specific antigen.

## ADVANCING PRACTICE

### What does this study add?

The study demonstrates that the 4Kscore test can significantly streamline prostate cancer screening by reducing unnecessary MRIs and biopsies resulting in lowered overdiagnosis of low-grade cancers, with only a minor risk of delaying some intermediate-grade cancer diagnoses. These results add further evidence for including new blood-based biomarkers as a complement to PSA testing and thus improve the balance between the harms and benefits of prostate cancer screening.

### Clinical Relevance

Optimisation of algorithms is a priority in current and future programs of organised prostate cancer screening. Whilst the utility of prostate MRI in men with elevated PSA is widely accepted, the role of “liquid” biomarkers as a reflex test is still undetermined. Investigators of the randomised GÖTEBORG-2 trial showed in an ancillary study including a relatively small subgroup of participants, that a “liquid” biomarker, such as the 4K score, performed as a reflex test in men with elevated PSA, had the benefits of reducing the number of MRI exams and biopsies, and the diagnosis of low-grade cancers at the cost of delaying the detection of higher-grade cancers in only a small proportion of men. Implications would be a cut to the increasing demand of prostate MRI in the community, and the use of a non-invasive, and potentially more convenient, liquid test. Associate Editor: Gianluca Giannarini, M.D.

### Patient Summary

In this study, 4Kscore, a blood-based biomarker, as a reflex test for men with elevated prostate-specific antigen (PSA), reduces the need for magnetic resonance imaging and biopsy. These results support the inclusion of new blood-based biomarkers in addition to PSA.
