# Supplementary material for: Performance of 4Kscore as a Reflex Test to Prostate-specific Antigen in the GÖTEBORG-2 Prostate Cancer Screening Trial
Source: Eur Urol. Author manuscript; Available in PMC 2025 Jan 21. (PMC11747930; doi:10.1016/j.eururo.2024.04.037)
Supplement: 1 [file NIHMS2046393-supplement-1.pdf]

Supplementary data

Method

Trial design and participants
In arm 1, all men with PSA ≥3.0 ng/mL also underwent 10-12 systematic core prostate biopsies in addition to the targeted biopsies of any MRI-positive lesions. In arm 2, participants with PSA ≥3.0 ng/mL underwent an MRI, but only men with a positive MRI were biopsied. Arm 3 was identical to arm 2, except that men with PSA ≥ 1.8 ng/mL underwent MRI. The MRI reading was performed by consensus reading by two experienced radiologists and classified according to the Prostate Imaging Reporting and Data System version 2 (PI-RADS)(1). Primary and secondary Gleason grade, and the International Society of Urological Pathology (ISUP) grading groups were annotated by one experienced pathologist.

Laboratory measurements

PSA measurement in the G2 study is described previously (2) . Up to 50 mL of blood was drawn and rapidly delivered to the biobank for processing and aliquoting for men included in the G2B study. Time from blood draw to the freezer was a median of 88 minutes (interquartile range [IQR] 77-104 minutes). Aliquots of plasma were shipped to Dr. Lilja's laboratory at Lund University where analyses of total, free, and intact PSA and hK2 were performed as described previously (3).

Pathological assessment

Those men detected only by a systematic biopsy in arm 1 were regarded as negative. In this analysis, biopsy results were restricted to targeted biopsies only. Biopsy cores harboring cancer were classified according to the Gleason grading system and based on the local pathologist´s assessment (2). Low-grade prostate cancer was defined as ISUP grade group 1 (Gleason 3+3), and high-grade cancer was defined as ISUP ≥2 (Gleason > 3+3)(4). Pathologic analysis was conducted blind to 4Kscore results and vice versa.

Statistical analysis

4Kscore was performed according to a prespecified algorithm(5) based on age and analysis of plasma concentrations of total PSA, free PSA, intact PSA, and human kallikrein-related peptidase 2 (hK2), giving the risk of diagnosis of ISUP≥2 prostate cancer. A cut-off of 7.5% was predefined for the assessment of the 4Kscore based on previous work (6).

References

1. Turkbey B, Rosenkrantz AB, Haider MA, Padhani AR, Villeirs G, Macura KJ, et al. Prostate Imaging Reporting and Data System Version 2.1: 2019 Update of Prostate Imaging Reporting and Data System Version 2. European urology. 2019;76(3):340-51.
2. Hugosson J, Mansson M, Wallstrom J, Axcrona U, Carlsson SV, Egevad L, et al. Prostate Cancer Screening with PSA and MRI Followed by Targeted Biopsy Only. The New England journal of medicine. 2022;387(23):2126-37.
3. Vaisanen V, Eriksson S, Ivaska KK, Lilja H, Nurmi M, Pettersson K. Development of sensitive immunoassays for free and total human glandular kallikrein 2. Clinical chemistry. 2004;50(9):1607-17.
4. Egevad L, Delahunt B, Srigley JR, Samaratunga H. International Society of Urological Pathology (ISUP) grading of prostate cancer - An ISUP consensus on contemporary grading. Apmis. 2016;124(6):433-5.
5. Parekh DJ, Punnen S, Sjoberg DD, Asroff SW, Bailen JL, Cochran JS, et al. A multi-institutional prospective trial in the USA confirms that the 4Kscore accurately identifies men with high-grade prostate cancer. European urology. 2015;68(3):464-70.
6. Braun K, Sjoberg DD, Vickers AJ, Lilja H, Bjartell AS. A Four-kallikrein Panel Predicts High-grade Cancer on Biopsy: Independent Validation in a Community Cohort. European urology. 2015.
