# Supplementary material for: Performance of 4Kscore as a Reflex Test to Prostate-specific Antigen in the GÖTEBORG-2 Prostate Cancer Screening Trial
Source: Eur Urol. Author manuscript; Available in PMC 2025 Jan 21. (PMC11747930; doi:10.1016/j.eururo.2024.04.037)
Supplement: 2 [file NIHMS2046393-supplement-2.pdf]

Supplementary Figure 1
Sensitivity, specificity, PPV, and NPV by different cut-offs of 4K score for diagnosis of any grade cancer (ISUP =1-5)

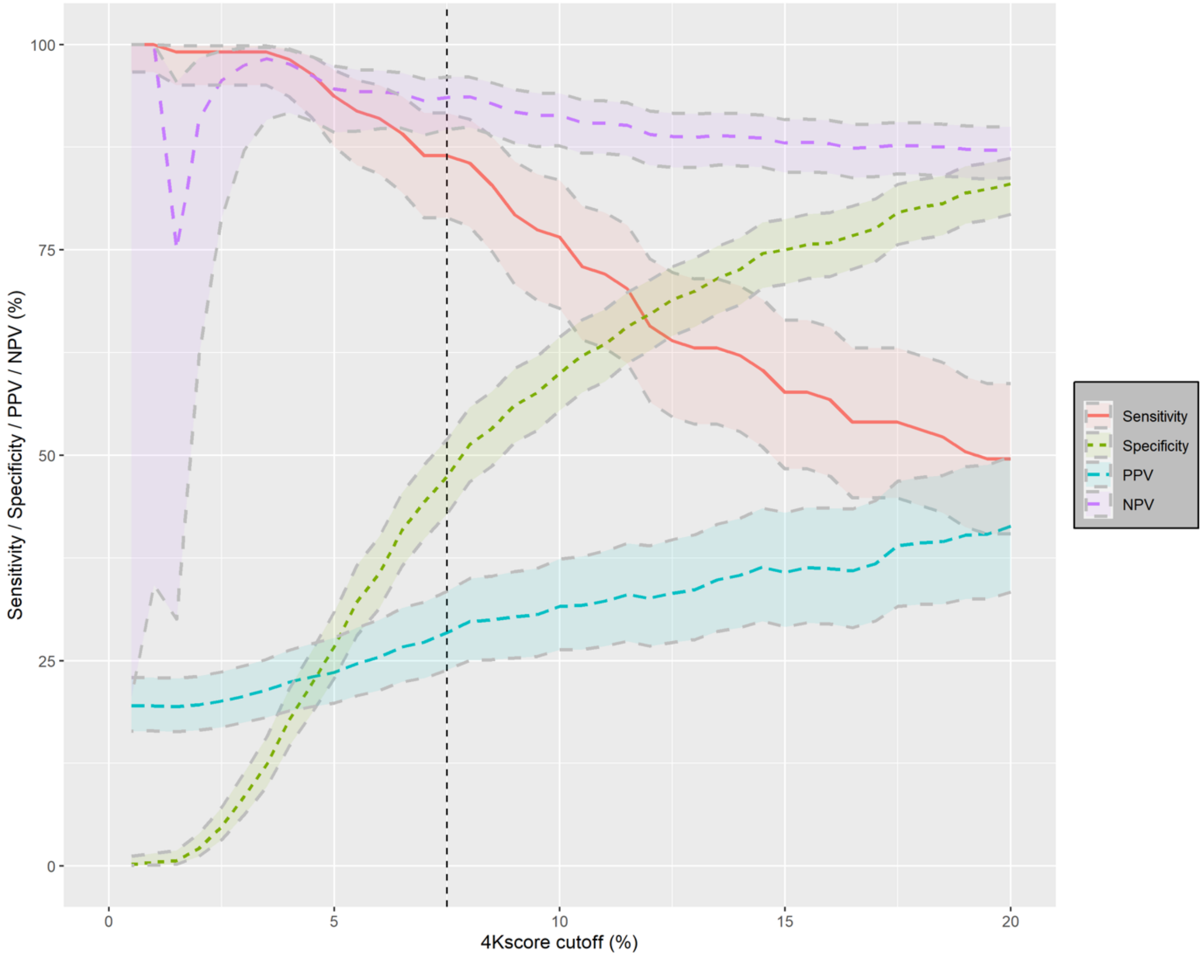

PPV; Positive predictive value, NPV; Negative predictive value, ISUP; International Society of Urological Pathology grade

Supplementary Figure 2

Decision curve analysis for 4Kscore as a reflex test before MRI for A) intermediate to high-grade prostate cancer (ISUP 2-5). The net benefit is defined as the threshold where men with positive MRI and prostate cancer are detected after taking harm of missing prostate cancer into account vs the risk of prostate cancer for which the patient or doctor chooses to perform an MRI. Net intervention (MRI examination) avoided for B) intermediate to high-grade prostate cancer (ISUP 2-5). Net reduction is defined as the threshold avoided MRI in men with negative MRI or benign biopsy, after taking harm of missing prostate cancer into account in performed MRIs vs. risk of prostate cancer for which patient/doctor would choose to perform MRI.

A

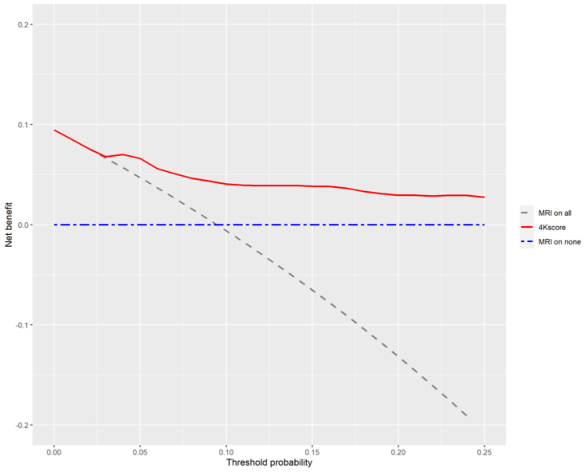

B

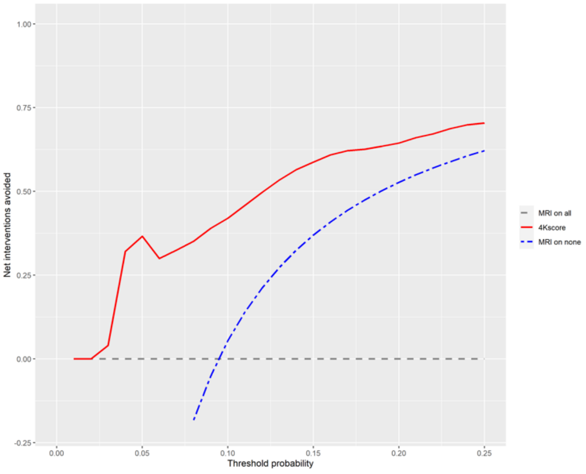

PI-RADS; Prostate Imaging Reporting and Data System version 2, MRI; Magnetic resonance imaging, ISUP; International Society of Urological Pathology grade,

Supplementary Table 1

Detection rates of cancers and saved MRIs and biopsies per 1,000 men with elevated PSA, using 4Kscore with a cut-off 5% as a reflex test in the diagnostic workup for men with a PSA ≥3.0 ng/ml followed by MRI and targeted biopsies of PI-RAD score ≥3

	PSA ≥3.0 ng/ml as trigger for diagnostic work up	PSA ≥ 3.0 ng/ml and 4Kscore ≥ 5% for diagnostic work up	
	Performed	Performed	Avoided/missed (%)
MRI	1000	772	228 (23%)
Negative MRI	658	485	173 (36%)
Positive MRI (PI-RADS ≥3)	342	287	55 (16%)
Targeted biopsies	342	287	55 (16%)
Benign biopsies	147	105	42 (29%)
ISUP 1	100	88	12 (12%)
ISUP ≥ 2	95	95	0 (0%)

PI-RADS; Prostate Imaging Reporting and Data System version 2, MRI; Magnetic resonance imaging, ISUP; International Society of Urological Pathology grade

Supplementary Table 2
4Kscores for men with elevated PSA (≥3.0 ng/ml) with a positive MRI (PI-RAD score ≥3) and ISUP grade of targeted biopsies.

	4Kscore			
	**0.0% - 5.0%**	**5.0% -7.5%**	**7.5% - 10%**	**10% - 94%**
	n = 31	n = 23	n = 28	n = 113
**ISUP (%)**				
**1**	7 (23)	6 (26)	8 (29)	36 (32)
**2**	0 (0.0)	2 (8.7)	2 (7.1)	36 (32)
**3**	0 (0.0)	0 (0.0)	1 (3.6)	7 (6.2)
**4**	0 (0.0)	0 (0.0)	0 (0.0)	4 (3.5)
**5**	0 (0.0)	0 (0.0)	0 (0.0)	2 (1.8)
NA	24 (75)	15 (65)	17 (61)	28 (25)

PI-RADS; Prostate Imaging Reporting and Data System version 2, MRI; Magnetic resonance imaging, ISUP; International Society of Urological Pathology grade

Supplementary Table 3
4Kscores for men with elevated PSA (≥3.0 ng/ml) for the PI-RAD score outcomes on MRI examination.

	4Kscore			
	**0.0% - 5.0%**	**5.0% -7.5%**	**7.5% - 10%**	**10% - 94%**
	n = 130	n = 103	n = 69	n = 269
**PI-RADS (%)**				
**1**	3 (2.3)	0 (0.0)	0 (0.0)	4 (1.5)
**2**	96 (74)	80 (78)	41 (60)	152 (57)
**3**	13 (10)	7 (6.8)	10 (15)	23 (8.6)
**4**	17 (13)	15 (15)	18 (26)	67 (25)
**5**	1 (0.8)	1 (1.0)	0 (0.0)	23 (8.6)

PI-RADS; Prostate Imaging Reporting and Data System version 2, MRI; Magnetic resonance imaging
